# Serum Lycopene Concentrations and Associations with Clinical Outcomes in a Cohort of Maternal-Infant Dyads

**DOI:** 10.3390/nu10020204

**Published:** 2018-02-13

**Authors:** Corrine Hanson, Elizabeth Lyden, Jeremy Furtado, Matthew Van Ormer, Kimberly White, Nina Overby, Ann Anderson-Berry

**Affiliations:** 1 College of Allied Health Professions Medical Nutrition Education, University of Nebraska Medical Center, Omaha, NE 68198-4045, USA; 2 College of Public Health, University of Nebraska Medical Center, Omaha, NE 68198-4375, USA; elyden@unmc.edu; 3 Department of Nutrition, Harvard School of Public Health, Boston, MA 02215, USA jfurtado@hsph.harvard.edu; 4 College of Medicine Pediatrics, University of Nebraska Medical Center, Omaha, NE 68198-1205, USA; Matthew.vanormer@unmc.edu (M.V.O.); kim.white@unmc.edu (K.W.); alanders@unmc.edu (A.A.-B.); 5 Department of Public Health, Sport and Nutrition, University of Agder, P.O. 422, 4606 Kristiansand, Norway; nina.c.overby@uia.no

**Keywords:** carotenoid, lycopene, pregnancy, neonatal growth

## Abstract

Oxidative stress has been associated with adverse neonatal outcomes, and many carotenoids, including lycopene, potentially have antioxidant properties. The objective of this analysis was to explore the associations between serum lycopene concentrations, including lycopene isomers, and maternal-newborn outcomes. Maternal and cord blood samples were collected in 180 mother-infant pairs. Serum of total lycopene as well as the *cis*- and *trans*-isomers concentrations were measured using HPLC (High Performance Liquid Chromatography). Descriptive statistics were calculated; Spearman coefficients were used to assess correlations between maternal and cord concentrations. The relationship between lycopene concentration and outcomes were evaluated with linear and logistic regression models, with adjustment for relevant confounders. A *p* ≤ 0.05 was considered statistically significant. Maternal and cord serum lycopene concentrations were positively correlated for total lycopene (*r* = 0.30, *p* < 0.0001), *cis*-lycopene (*r* = 0.29, *p* = 0.0002); and *trans*-lycopene (*r* = 0.32, *p* < 0.0001). Maternal concentrations of *cis*-lycopene were significantly lower in mothers whose infants developed respiratory distress syndrome compared to those who did not (0.336 ± 0.171 vs. 0.445 ± 0.238 µmol/L, *p* = 0.04) and also in mothers whose babies were admitted to the newborn intensive care unit compared to those who were not (0.380 ± 0.202 vs. 0.458 ± 0.244 µmol/L, *p* = 0.04). Conversely, cord concentrations of *trans*-lycopene were significantly higher in infants who developed RDS (Respiratory Distress Syndrome) (0.023 ± 0.012 vs. 0.016 ± 0.012, *p* = 0.007 for RDS vs. no RDS), and a similar pattern was seen NICU admission (0.023 ± 0.016 vs. 0.015 ± 0.009 µmol/L for NICU (Newborn Intensive Care Unit) admission vs. no NICU admission, *p* = 0.007). Maternal concentrations of total and *cis*-lycopene were positively associated with infant birth weight, length and head circumference after adjustment for relevant confounders. As serum carotenoids, including lycopene, are modifiable by diet, future research determining the clinical impact of these compounds is warranted.

## 1. Introduction

Oxidative stress results from an imbalance between the production of potentially harmful reactive oxygen species (ROS) and antioxidants and their repair processes [1,2,3,4]. During pregnancy, an increase in oxidative stress can raise the risk of many adverse outcomes, including gestational diabetes, preeclampsia, pregnancy-induced hypertension, and intrauterine growth restriction [1,2,5,6,7,8]. Dietary antioxidants, including carotenoids, could potentially play a role in decreasing oxidative stress through their capacity to inhibit reactive oxygen species and scavenge free radicals [9,10] and therefore represent potentially modifiable exposures in decreasing oxidative stress during pregnancy.

Carotenoids are natural pigments synthesized by plants and microorganisms to serve as light- ablating compounds during photosynthesis and protection of cells against photosensitization [11]. More than 700 carotenoids have been characterized and about 60 of these carotenoids are found in the human diet [12,13]. Major dietary classes include carotenes and xanthophylls, such as lutein and β-cryptoxanthin [11,12]. Diet is one determinant of plasma concentrations of carotenoids [14], and up to 95% of total plasma carotenoid concentration is composed of six compounds: β-carotene, α-carotene, β-cryptoxanthin, lutein, zeaxanthin, and lycopene [12]. Lycopene is a very prominent carotenoid in human plasma and tissue and may comprise up to 50% of the total carotenoids content of the human body [11,15].

Lycopene is a non-pro-vitamin A carotenoid, but has demonstrated potential antioxidant activity [4]. Lycopene in vitro has been shown to be 2–10 times more effective at quenching reactive oxygen species than other nutritional antioxidants, including β-carotene or α-tocopherol [4], given the relatively low concentrations of lycopene in human plasma; however, the health effects of lycopene seen in epidemiological trials may be due to other actions, such as alteration of gene expression by metabolic products of lycopene [4]. Lycopene may also play a role in immune development [16,17], and protection against inflammatory diseases [4,18,19]. Specific to maternal-child outcomes, increases in serum concentrations of lycopene have been associated with a decrease in rates of pre-eclampsia [15,20] and fetal growth restriction [15,20,21], although these results are inconsistent, and may not necessarily be related to anti-oxidant effects [22,23,24]. Improvements in analytical technology in recent years have led to the discovery that lycopene in human blood and tissue is distributed among 10–20 different isomers [25]. Typical food sources of lycopene contain 60–95% *trans*-lycopene [25], however; tissue and plasma have higher concretions of *cis*-lycopene, possibly due to post-absorptive *trans*-to-*cis*-lycopene isomerization [26]. However, the transfer from mother to fetus of these isomers, or differing impacts on maternal-newborn outcomes, has rarely been investigated. Therefore, the objective of this analysis was to explore the associations between serum lycopene concentrations, including lycopene isomers, and maternal-newborn outcomes.

## 2. Methods

This study was an exploratory analysis of serum lycopene concentrations that were obtained as part of a cross-sectional study evaluating the retinol status of 180 maternal-infant pairs recruited at the time of delivery in a Midwestern United States academic medical center. Detailed results of that study have been published elsewhere [27]. After obtaining Institutional Review Board approval, and informed consent from all participants, samples of both cord and maternal blood were collected at the time of delivery from mothers who consented to participate. Exclusion criteria included congenital abnormalities, gastrointestinal liver, or kidney disease, or inborn errors of metabolism in the infant or the mother. Samples were protected from heat and light and processed and frozen at −80 °C immediately.

### 2.1. Evaluation of Serum Concentrations

Analysis of samples was performed at the Biomarker Research Institute at the Harvard School of Public Health. Concentrations of total, *cis*-, and *trans*-lycopene in plasma samples were measured as described by El-Sohemy et al. [28]. Plasma samples (250 μL) were mixed with 250 μL ethanol containing 10 μg/mL *rac*-Tocopherol (Tocol) as an internal standard, extracted with 4 mL hexane, evaporated to dryness under nitrogen, and reconstituted in 100 μL ethanol-dioxane (1:1 *v*/*v*) and 150 μL acetonitrile. Samples were quantitated by high-performance liquid chromatography (HPLC) on a Restek Ultra C18 150 mm × 4.6 mm column, 3 μm particle size encased in a column oven (Hitachi L-2350, Hitachi, San Jose, CA, USA) to prevent temperature fluctuations, and equipped with a trident guard cartridge system (Restek, Corp., Bellefonte, PA, USA). A mixture of acetonitrile, tetrahydrofuran, methanol, and a 1% ammonium acetate solution (68:22:7:3) was used as mobile phase at a flow rate of 1.1 mL/min, with a Hitachi L-2130 pump in isocratic mode, a Hitachi L-2455 diode array detector (300 nm and 445 nm), and a Hitachi L-2200 auto-sampler with water-chilled tray. The Hitachi System Manager software (D-2000 Elite, Version 3.0, Hitachi, Schaumburg, IL, USA) was used for peak integration and data acquisition. The minimum detection limits (MDLs) in plasma are (μg/L) 5.44 for lycopene. The between-run coefficients of variation (CV) for lycopene are under ~5% in plasma.

Internal quality control is monitored with four control samples analyzed within each run. These samples consist of two identical high-level plasmas and two identical low-level plasmas. Comparison of data from these samples allows within-run and between-run variation estimates. In addition, external quality control is monitored by participation in the standardization program for carotenoid analysis from the National Institute of Standards and Technology USA.

### 2.2. Other Study Variables

Maternal demographic information included age, race, ethnicity, marital status, smoking status (yes/no), payor sources (public vs. private), and pre-pregnancy Body Mass Index (BMI). Clinical maternal outcomes evaluated included mode of delivery (vaginal vs. Cesarean-section), and occurrence of gestational diabetes or pre-eclampsia. Nutrient intake information was collected from the mothers using the Willett Food Frequency Questionnaire. The FFQ (Food Frequency Questionnaire) allows for analysis of absolute nutrient intake values from foods and as well as a wide variate of supplements. FFQs were analyzed by trained personnel in the Harvard University Department of Nutrition.

Infant clinical data collected at the time of delivery included gestational age, race, birth weight, length, and head circumference. Infants were weighed by nursing staff daily on a gram scale, and head circumference and length in centimeters were recorded weekly. Infant growth curve percentile rankings were calculated and plotted electronically for each recorded anthropometric measurement. Additional newborn outcomes included a clinical diagnosis of Respiratory Distress Syndrome (RDS) and admission to the Newborn Intensive Care Unit (NICU).

### 2.3. Statistical Analysis

Descriptive statistics were performed for all variables. Means, standard deviations, counts and percentages were used to summarize the data. Spearman correlations coefficients were used to look at the association between maternal and cord lycopene concentrations. Independent sample *t*-tests were used to compare continuous measures between dichotomous groups. Linear and logistic regression models were used to evaluate the associations between the independent variable (lycopene concentrations) with clinical outcomes, with adjustment for relevant confounders. The potential confounding variables of gestational age, smoking, and race were chosen based on associations with outcomes of interest. A *p*-value of ≤0.05 was considered statistically significant.

## 3. Results

### 3.1. Baseline Characteristics

Data from one hundred eighty mothers and 173 infants was available for analysis in this study. Seven cord blood samples were not available for analysis as they were required for clinical purposes; however, we chose to analyze all available samples. Mean maternal age was 28.7 years ± 5.6; mean maternal pre-pregnancy BMI was 27.1 kg/m^2^ ± 6.68. Mean birth corrected gestational age was 38 weeks ± 3.07. In terms of race/ethnic group, 58% of mothers were white, 14.8% were African-American, 12% were Hispanic, and 1.6% were Asian/Pacific Islander. Most of the mothers were non-smokers (84.13%), and 14.8% were current smokers. The mean serum total lycopene of the maternal cohort was 0.874 ± 0.462 µmol/L; the mean serum total lycopene of the infant (cord) cohort was 0.041 ± 0.048 µmol/L. Mean dietary lycopene intake in the maternal cohort was 10.57 µmol/day. Baseline characteristics of the maternal and infant cohort are shown in Table 1.

### 3.2. Serum Lycopene Concentrations

Total lycopene and its isomers were significantly lower in cord blood when compared to maternal blood (Table 1). Mean total lycopene in the cord serum was 5% of the total serum value in the maternal samples (0.041 ± 0.048 vs. 0.874 ± 0.462 µmol/L). Similarly, the mean *cis*-lycopene in the cord serum was 5% of the maternal values (0.023 ± 0.031 vs. 0.432 ± 0.234 µmol/L) and cord *trans*-lycopene concentrations were 4% of the maternal concentrations (0.018 ± .0018 vs. 0.442 ± 0.236 µmol/L). Compared to non-white mothers, white mothers had higher concentrations of total lycopene (0.929 ± 0.50 vs. 0.781 ± 0.384 µmol/L, *p* = 0.03), *cis*-lycopene (0.460 ± 0.257 vs. 0.385 ± 0.186 µmol/L, *p* = 0.03), and *trans*-lycopene (0.469 ± 0.251 vs. 0.396 ± 0.205 µmol/L, *p* = 0.04). After adjustment for other cofounding socioeconomic factors of smoking, maternal age, payer source, and geographic area, total lycopene and *cis*-lycopene remained significantly higher in white mothers than non-white mothers (*p* = 0.04 and 0.04, respectively).

Maternal *cis*-lycopene concentrations were significantly associated with gestational age of the infant at birth (*r* = 0.16, *p* = 0.03), however total and *trans*-lycopene were not associated with gestational age. Serum lycopene concentrations were not associated with pre-pregnancy BMI of the mother.

### 3.3. Maternal-Cord Transfer of Lycopene Isomers

Maternal and cord serum lycopene concentrations were positively correlated for total lycopene (*r* = 0.30, *p* < 0.0001), *cis*-lycopene (*r* = 0.29, *p* = 0.0002); and *trans*-lycopene (*r* = 0.32, *p* < 0.0001).

### 3.4. Maternal Lycopene Intake and Serum Correlations

No significant associations were seen between maternal lycopene intake and serum concentrations of total lycopene (*r* = 0.13, *p* = 0.14), *cis*-lycopene (*r* = 0.12, *p* = 0.19) or *trans*-lycopene (*r* = 0.14, *p* = 0.10).

### 3.5. Maternal-Infant Outcomes and Lycopene Concentrations

Maternal *cis*-lycopene concentrations were significantly lower in mothers whose infants developed RDS (0.336 ± 0.171 vs. 0.445 ± 0.238 µmol/L for RDS vs. no RDS, *p* = 0.04), and maternal concentrations of *cis*-lycopene were also lower in mothers whose babies were admitted to the NICU (0.380 ± 0.202 vs. 0.458 ± 0.244 µmol/L for NICU admission vs. no NICU admission, *p* = 0.04). Conversely, cord concentrations of *trans*-lycopene were significantly higher in infants who developed RDS (0.023 ± 0.012 vs. 0.016 ± 0.012 µmol/L, *p* = 0.007 for RDS vs. no RDS), and a similar pattern was seen for cord *trans*-lycopene levels of infants who were admitted to the NICU (0.023 ± 0.016 vs. 0.015 ± 0.009 µmol/L for NICU admission vs. no NICU admission, *p* = 0.007). Infants delivered via Cesarean section (C-section) had higher cord concentrations of trans-lycopene when compared to infants delivered vaginally (0.019 ± 0.011 vs. 0.016 ± 0.013 µmol/L for C-section vs. vaginal delivery, *p* = 0.05). Infants who were admitted to the NICU also had significantly higher total lycopene (0.044 ± 0.033 vs. 0.035 ± 0.024 µmol/L, for yes vs. no NICU admit; *p* = 0.03). There was no significant difference in maternal or cord lycopene or its isomers and the presence of maternal diabetes (0.880 ± 0.458 vs. 0.778 ± 0.470 µmol/L total lycopene for no gestational diabetes vs. yes, *p* = 0.15) or the occurrence of preeclampsia (0.866 ± 0.468 vs. 0.882 ± 0.358 µmol/L total lycopene for no preeclampsia vs. yes, *p* = 0.58).

### 3.6. Infant Growth Parameters at Birth and Lycopene

In the univariate analysis, maternal total, *cis*, and *trans*-lycopene concentrations were positively associated with multiple measures of infant growth at birth (Table 2). Conversely, inverse associations were seen between cord concentrations of trans-lycopene and birth weight (*r* = −0.18, *p* = 0.02) and length (*r* = −0.21, *p* = 0.006). 

After adjustment for the confounding factors of gestational age, maternal smoking, and race, significant positive relationships were maintained between maternal total lycopene and birth weight (*p* = 0.04), birth weight percentile ranking (*p* = 0.008), head circumference (*p* = 0.009), and birth length percentile ranking (*p* = 0.02). Maternal concentrations of *cis*-lycopene remained positively associated with birth weight (*p* = 0.040), birth weight percentile ranking (*p* = 0.006), head circumference (*p* = 0.02), head circumference percentile ranking (*p* = 0.05), and birth length percentile ranking (*p* = 0.01). Maternal *trans*-lycopene concentrations were positively associated with birth weight (*p* = 0.04), birth weight percentile ranking (*p* = 0.01), head circumference (*p* = 0.007), and birth length percentile ranking (*p* = 0.02). Cord concentrations of *trans*-lycopene retained an inverse association with birth weight (*p* = 0.04). These relationships are shown in Table 3.

## 4. Discussion

Our study found that maternal serum concentrations of total lycopene and the *cis*-isoform were positively associated with most growth parameters in the newborn. Conversely, serum cord concentrations of *trans*-lycopene showed an inverse association with newborn birth weight. Vitamin A deficiency has been associated with growth failure [21,29]; therefore, investigations often focus on carotenoids with pro-vitamin A activity, such as β-carotene. The roles of major non-pro-vitamin A compounds such as lycopene are not well established. Several studies of lycopene and growth have been conducted in developing nations, where the incidence of intrauterine growth restriction (IUGR) and childhood wasting can be high. Results of a study in Uganda showed that low concentrations of lycopene were associated with both ponderal and linear growth failure in HIV-infected infants [30]. This association has also been shown in healthy infant; one study of 173 healthy infants in Malawi showed a positive association between non-pro-vitamin A carotenoids infant serum and weight for age at 12 months after adjustment for confounders; however, lycopene was not reported separately [21]. It is worth noting these studies were conducted in different countries, where lycopene intakes may be significantly different than those of American women. Additionally, the overall nutritional status and overall diet quality of these women could be different than women in the United States, making it important to study these relationships in American women. In a rare study evaluating serum carotenoid levels and infant growth in a developed nation, maternal carotenoid levels at 24–26 weeks gestation, including β-cryptoxanthin, β-carotene, but not lycopene, were associated with small for gestational age births in a cohort of maternal-infant pairs in Montreal, Quebec, Canada [23]. 

Several randomized controlled trials of lycopene supplementation in pregnant women have been conducted in underdeveloped nations. In one such study, a cohort of women in India received 2 mg twice daily lycopene supplementation during their pregnancy [15]. The findings of this study reported that mean fetal weight was significantly higher in the supplemented group compared to the placebo, and the incidence of intrauterine growth restriction (IUGR) was lower [15]. Another study of pregnant women in India who received either 2 mg of lycopene twice daily or placebo showed that the risk of IUGR was reduced by 80% in the supplemented group [22]. These results, however, remain controversial, with a 2009 study of 2 mg lycopene once daily in pregnant women in India demonstrating an increase in the incidence of low birth weight infants [24]. It is of note that the dose of lycopene in this study was half the dose of the earlier studies that demonstrated a positive impact of lycopene on growth parameters.

Our results now extend these findings to demonstrate an association between maternal lycopene levels and growth parameters in infants in the Midwest region of the United States. Furthermore, to our knowledge, we are the first to report the individual isoforms of lycopene, and show opposing associations of the *cis*-isoform in maternal serum and the *trans*-isoform in cord serum with growth outcomes. Numerous studies have suggested that isomerization from the *trans* to *cis* forms increases the bioavailability of lycopene, and hence its biological effects [19,31,32,33,34]. However, there is no evidence to suggest that the *cis*-lycopene isomer has any additional health benefits over the *trans*- form. Kinetic studies in adults have shown that the proportion of *cis*-lycopene isomers increase over time, and *trans*-lycopene isomers have a shorter half-life than *cis*-lycopene isomers [25,26]; however, the kinetics of lycopene isomers have not been studied in neonates. Therefore, we could speculate that the beneficial effects of lycopene on growth are more profound after the conversion of trans-lycopene to the more biologically active *cis*-lycopene, and mechanisms by which this conversion is performed may be immature in a newborn infant.

Although our study did not show any associations between serum lycopenes and pre-eclampsia, previous studies have been conducted to evaluate this relationship with mixed results. Sharma et al. demonstrated lower risk of pre-eclampsia with lycopene supplementation in the second trimester [15], however other studies have failed to confirm these findings [22,24]. Both of the studies showing no effect of lycopene supplementation had a small sample size and were conducted in samples with different health status (i.e., high and low risk for preeclampsia). It is possible that the overall low incidence of pre-eclampsia in our cohort (8%) obscured any significant results.

It has been hypothesized that protective antioxidant systems are deficient in pre-eclampsia and that oxidative stress could lead to placental infarction, endothelial dysfunction, and restricted fetal growth [20]. Although it is possible that the low incidence of pre-eclampsia in our study did not allow us to determine any associations with that condition, we did see associations with restricted fetal growth, and our results also show associations between maternal lycopene levels and the incidence of RDS, another disease which may be related to oxidative stress [35]. It is also possible that without supplementation, the serum lycopene levels in our cohort were not high enough to exert an effect on pre-eclampsia. Recent studies propose that this antioxidant concept is controversial because of the relatively high carotenoid concentrations needed to elicit these antioxidant effects [4,17]. Serum lycopene levels of the supplemented and non-supplemented cohorts are not reported in the pre-eclampsia trials described earlier for comparison with our cohort.

Similar to other studies of carotenoids, our study found that the lycopene status of the newborn was directly dependent on the serum concentrations of the mother, and that cord blood concentrations are several times lower [29,36,37,38,39]. This has been attributed to a decreased capacity for transport for lipophilic substances due to low levels of circulating lipoproteins in fetal blood [37]. Other possibilities for this difference exist, including uptake and metabolism by the placenta. It has been proposed that the decreased levels of pro-vitamin A compounds in newborn serum may be due to intensive conversion of these compounds to retinol by the fetus for storage in the liver [12], but this mechanism would not seem to apply to non-pro vitamin A carotenoids. 

Our study did not find an association between lycopene intake and serum lycopene concentrations in pregnancy women at the time of delivery. However, the results of the Norwegian Mother and Child Cohort study (MoBa) has confirmed that the plasma concentrations of carotenoids are a strict function of intakes of fruits and vegetables [14] and other studies have demonstrated serum lycopene levels are directly correlated with intakes of food high in lycopene [19]. In contrast to other carotenoids, dietary lycopene is contained in relatively few foods, including tomatoes, guava and watermelon, and tomatoes account for over 80% of the lycopene in American diets [34]. Dietary intake methodology such as Food Frequency Questionnaires, which was what was used in this study, should be effective at capturing the intakes of food such as these which may not be a daily occurrence.

The effects of supplementing individual compounds, such as lycopene, merit much consideration prior to implementation. Clinical trials with other single small molecules, like vitamin E, vitamin C, or β-carotene have largely been disappointing [40], and likewise, laboratory experiments using single antioxidants have shown them to be largely ineffective in ameliorating hyperoxia-induced injury [3,41]. An imbalance between different antioxidants can potentially result in tissue damage [3,42]. Antioxidant defense systems are multifaceted, thus supplementation with a single molecule, unless a deficiency is present, is likely to have little effect on the development of a disease or adverse outcome. On the other hand, a portfolio of small molecules, such as those found in fruits and vegetables, may provide significant protection [4].

### Limitations

Our study reports associations between maternal-infant outcomes and serum lycopene; however, markers of other antioxidants and measures of oxidative stress would also be of interest in order to more fully understand the balance of factors that contribute to a state of oxidative stress. Additionally, the correlations seen in our study may not be of the magnitude that would be required in order so see significant clinical impacts. It is possible that serum lycopene concentrations, or tomato consumption, may just be a surrogate marker for an overall healthy lifestyle. Our small sample size made it difficult to assess for the many confounders that could be associated with fetal growth and outcomes, however our choices of gestational age and smoking are likely to have a large confounding effect. We did see significant associations between lycopene status as both RDS and NICU admission, however many NICU admissions are due to RDS. Our study was exploratory in nature and utilized multiple statistical comparisons, therefore the results may be best interpreted as hypothesis-generating. 

## 5. Conclusions

Lycopene, a carotenoid with no pro-vitamin A activity but powerful antioxidant potential, was correlated in maternal-infant pairs and associated with newborn outcomes. As these lycopene is modifiable by maternal diet, more research into the impact of lycopene and other carotenoids on pregnancy and neonatal outcomes could have a beneficial impact on maternal and infant health.

## Figures and Tables

**Table 1:** Demographic and clinical characteristics of the maternal and infant cohort.

Maternal/Infant Characteristic	*N*	Mean (SD)
Maternal Age (years)	189	28.7 (5.6)
Maternal BMI (pre-pregnancy) (kg/m^2^)	112	27.1 (6.6)
Infant Corrected Gestational Age at birth (weeks)	189	38.04 (3.1)
Infant Birth Anthropometrics:		
Birth Weight (g)	189	3109.8 (783.4)
Birth Length (cm)	189	48.43 (4.7)
Birth head circumference (cm)	189	33.5 (2.8)
Maternal serum concentrations (µmol/L)		
Total lycopene	180	0.874 (0.462)
*Cis*-lycopene	180	0.432 (0.234)
*Trans*-lycopene	180	0.442 (0.236)
Infant serum (cord) concentrations (µmol/L)		
Total lycopene	173	0.041 (0.048)
*Cis*-lycopene	173	0.023 (0.031)
*Trans*-lycopene	173	0.018 (0.018)
Maternal Lycopene intake (µmol/day)	132	10.57 (7.91)
	*N*	%
Maternal Race		
White	111	58.7
African-American	28	14.8
Hispanic	24	12.7
Asian/Pacific Islander	3	1.6
Other	23	12.7
Smoking Status		
Current smokers	28	15
Former/Never Smokers	148	85
Mode of delivery		
Cesarean Section	63	35
Vaginal Delivery	114	65
Infant Gender		
Male	96	51
Female	93	49
Maternal Pre-eclampsia		
Yes	15	7.9
No	175	92.1
Newborn NICU admission		
Yes	73	38.4
No	117	61.6
Newborn RDS diagnosis		
Yes	28	14.8
No	162	85.2

**Table 2:** Univariate correlations between maternal and cord serum total, *cis*-, and *trans*-lycopene and infant growth variables.

Growth Variable	Maternal Serum			Cord Serum		
	Total Lycopene	*Cis*-Lycopene	*Trans*-Lycopene	Total Lycopene	*Cis*-Lycopene	*Trans*-Lycopene
	Spearman Correlation Coefficient *p*-Value					
Birth weight (g)	0.14	0.19	0.12	−0.13	−0.07	−0.18
	0.05	0.01	0.11	0.10	0.34	0.02
Birth weight percentile	0.17	0.19	0.16	−0.027	−0.02	−0.03
	0.02	0.02	0.03	0.74	0.83	0.68
Birth Length (cm)	0.11	0.15	0.09	−0.15	−0.09	−0.21
	0.13	0.04	0.20	0.05	0.23	0.006
Birth length percentile	0.14	0.15	0.14	−0.09	−0.07	−0.11
	0.05	0.05	0.05	0.2213	0.3383	0.1483
Birth head Circumference (cm)	0.15	0.20	0.12	0.01	0.06	0.07
	0.04	0.008	0.10	0.95	0.44	0.37
Birth head circumference percentile	0.16	0.17	0.15	0.10	0.11	0.08
	0.04	0.03	0.04	0.18	0.15	0.30

**Table 3:** Results of multivariate analysis for maternal serum concentrations of total lycopene and infant growth outcomes at birth after adjustment for gestational age, race, and maternal smoking (non-significant results not shown).

Growth Variable (at Birth)	Maternal Total Lycopene		Maternal *Cis*-Lycopene		Maternal *Trans*-Lycopene	
	β	*p*-Value	β	*p*-Value	β	*p*-Value
Birth Weight *	0.29	0.03	0.59	0.03	0.53	0.04
Birth Weight percentile **	0.02	0.008	0.05	0.006	0.04	0.01
Head Circumference (cm) *	0.001	0.009	0.003	0.01	0.003	0.007
Head Circumference ** percentile	-	-	0.04	0.05	-	-
Length *	0.001	0.20	0.002	0.18	0.002	0.24
Length percentile **	0.02	0.01	0.05	0.01	0.04	0.02
	**Cord Total Lycopene**		**Cord *Cis*-Lycopene**		**Cord *Trans*-Lycopene**	
Birth Weight *	-	-	-	-	−10.3	0.04
Birth Weight percentile **	-	-			-	-
Head Circumference (cm) *	-	-	-	-	-	-
Head Circumference percentile **	-	-	-	-	-	-
Length *	-	-	-	-	-	-
Length percentile **	-	-	-	-	-	-

* Models adjusted for gestational age and smoking; ** Models adjusted for smoking (percentile rankings are gestational age adjusted measures).
